# Experiencia de cinco años en el manejo de endocarditis infecciosa complicada en un centro de referencia nacional

**DOI:** 10.47487/apcyccv.v1i3.77

**Published:** 2020-09-30

**Authors:** Gracia del Carmen Polo Lecca, Lucio Torres Villacorta, Jhoel Yarahuaman Mora, Carlos Lobato Jerí, Edwin Uribe Badillo

**Affiliations:** 1 Servicio de Cardiología Clínica. Instituto Nacional Cardiovascular INCOR. Lima-Perú*. Lima Perú; 2 Servicio de Cardiología. Hospital Nacional Guillermo Almenara Irigoyen, Lima, Perú. Lima Perú; 3 Instituto Nacional Cardiovascular INCOR. Lima, Perú. Lima Perú

**Keywords:** Endocarditis, Mortalidad, Cirugía, Endocarditis, Mortality, Surgery

## Abstract

**Objetivo.:**

Evaluar las características epidemiológicas, clínicas, ecocardiográficas, microbiológicas y complicaciones de pacientes con endocarditis infecciosa complicada, atendidos en un hospital de referencia peruano.

**Material y métodos.:**

Se realizó un estudio retrospectivo, descriptivo, de pacientes con diagnóstico de endocarditis infecciosa atendidos en el Instituto Nacional Cardiovascular (INCOR) entre los años 2012 y 2016; se recolectaron sus variables clínicas, imagenológicas y de laboratorio.

**Resultados.:**

Se incluyeron 59 casos, con predominio del sexo masculino (66,1%) y mediana de edad de 50 años (RIQ 37-62). Las comorbilidades más frecuentes fueron la cardiopatía congénita (42,3%) y la presencia de válvula protésica (23,7%). El signo más frecuente en el examen físico fue la fiebre (69,49%) y el síntoma más común fue la disnea (52,5%). La proporción de hemocultivos positivos fue del 55,9%, y en el 51,5% de estos el patógeno aislado fue *Staphylococcus* spp. La válvula más afectada fue la aórtica (72,8%) y el hallazgo más frecuente por ecocardiografía fue la presencia de vegetaciones (91,5%). Las complicaciones más comunes fueron el bloqueo auriculoventricular (28,8%) y la falla cardiaca (22%). La mortalidad global intrahospitalaria fue del 20,3%.

**Conclusión.:**

La endocarditis infecciosa continúa siendo una patología desafiante, nuestros resultados clínico-epidemiológicos son comparables a los encontrados internacionalmente, los que reflejan el cambio que está sufriendo esta patología tanto en su microbiología como en su epidemiologia. Sin embargo, a pesar de los avances en diagnóstico y tratamiento, la mortalidad se mantiene sin cambio.

## Abstract (en)

**Objective.:**

To evaluate the epidemiological, clinical, echocardiographic, microbiological characteristics and complications of patients with complicated infective endocarditis (IE) in a Peruvian refence hospital.

**Material and methods.:**

A retrospective, descriptive study was carried out reviewing the medical records of patients diagnosed with IE treated at Instituto Nacional Cardiovascular-INCOR between years 2012 and 2016; collecting clinical, imaging and laboratory variables.

**Results.:**

59 cases were included, predominantly males (66.1%) and the median age was 50 years (IQR 37-62). The most frequent comorbidities were congenital heart disease (42.3%) and the presence of a prosthetic valve (23.7%). The most frequent sign found in the physical examination was fever (69.49%) and the most common symptom was dyspnea (52.5%). The proportion of positive blood cultures was 55.9%, and in 51.5% of these the isolated pathogen was Staphylococcus spp. The most affected valve was the aortic (72.8%), the most frequent finding by echocardiography was the presence of vegetations (91.5%). The most common complications were atrioventricular block (28.8%) and heart failure (22%). Overall, in-hospital mortality was 20.3%.

**Conclusion.:**

IE continues to be a challenging pathology, our clinical-epidemiological results are comparable to those found internationally, which reflect the change in the microbiology and in its epidemiology. However, despite advances in diagnosis and treatment, mortality remains unchanged.

La endocarditis infecciosa (EI) es una enfermedad poco común, con una incidencia que varía entre 4,9 ^1^ a 7,8 casos por 100 000 personas-año ^2^; sin embargo, tiene una alta tasa de morbilidad y mortalidad dentro de las enfermedades cardiovasculares. Se estima una mortalidad aproximada de 30% al año en Estados Unidos ^2^^,^^3^, lo cual representa un desafío permanente para la medicina moderna, en la que, a pesar de los crecientes avances tecnológicos, nuevos métodos microbiológicos y de diagnóstico en imágenes, el criterio clínico médico es determinante y fundamental ante la sospecha de dicha patología. 

La epidemiología de esta entidad ha cambiado, así, se reporta mayor media de edad, menor proporción de pacientes con cardiopatía congénita predisponente, diferente microbiología y mayor resistencia antibiótica ^2^^-^^5^; lo que, sumado a la heterogénea forma de presentación y el retraso en el diagnóstico temprano en países subdesarrollados como el nuestro, implica una significativa mayor tasa de complicaciones y mortalidad ^6^^-^^8^.

En el Instituto Nacional Cardiovascular (INCOR), que es un hospital de referencia de patologías cardiovasculares clínicas y quirúrgicas complejas a nivel nacional, hemos observado un incremento significativo de pacientes con EI complicada en los últimos años; por lo tanto se realiza este estudio con el objetivo de conocer las características clínicas, hallazgos laboratoriales, ecocardiográficos y las complicaciones intrahospitalarias de este grupo de pacientes.

## Material y métodos

Estudio retrospectivo, descriptivo de pacientes que ingresaron al INCOR de Lima-Perú, con el diagnóstico de EI entre enero 2012 a diciembre 2016. Se incluyeron a los pacientes con diagnóstico de EI complicada con falla cardiaca, infección persistente o riesgo de embolización. Para la confirmación de EI se utilizaron los criterios de Duke modificados ^6^. Se excluyeron pacientes con historias incompletas y casos de endocarditis de etiología no infecciosa.

Se definió falla cardiaca como la presencia de signos de congestión pulmonar persistente, edema pulmonar o choque cardiogénico, causado por insuficiencia mitral o aortica severa, obstrucción o fístula ^(^^6^. Riesgo de embolización se definió como la presencia de vegetaciones >10 mm, vegetaciones móviles, de localización preferente en válvula mitral, embolismo previo, y EI multivalvular ^6^; en tanto que la Infección persistente se definió como la persistencia de fiebre o de hemocultivos positivos después de 7 a 10 días de tratamiento antibiótico ^6^. Para la definición de lesión renal se usó la clasificación de AKIN (Acute Kidney Injury Network), siendo positiva para este estudio la presencia de AKIN I o mayor.

El proceso de recoleción de datos incluyó la revisión de los registros de ingreso de pacientes en los servicios de emergencia, cirugía cardiovascular y patología, luego se revisaron sus historias clínicas y se llenó la ficha de recolección de datos.

Se analizaron los datos con el programa STATA 16. Se realizó un análisis descriptivo mediante porcentajes y frecuencia para las variables nominales, y en las variables numéricas se utilizó la prueba Shapiro Wilk para determinar la distribución normal de las variables y, de acuerdo con ello, se usó la media y desviación estándar si la distribución fue normal, o la mediana y rango interquartil (RIQ) si no tenía distribución normal.

## Resultados

Se identificaron 82 historias clínicas con el diagnóstico de ingreso de EI, de las cuales 12 fueron excluidas por estar incompletas, 8 no tuvieron diagnostico final de EI y 3 no fueron encontradas en el archivo; finalmente, al estudio ingresaron 59 casos de EI complicada con falla cardiaca (40,7%), infección persistente (33,9%) y riesgo embolico (15,4%). La proporción varón : mujer fue de 1,9:1. Las características clínicas y laboratoriales de nuestra población de estudio se describen en la **(**Tabla 1**).**

**Tabla 1:** Características clínicas y laboratoriales de pacientes con EI complicada

Características generales	
Edad (años)	50 (37-62) *
Sexo masculino	39 (66,1%)
Localización de EI	
Aortica	43 (72,8%)**
Mitral	23 (38,9%)**
Tricúspide	4 (6,7%)**
Pulmonar	4 (6,7%)**
Tipo de EI	
Monovalvular	45 (76,2%)**
Multivalvular	14 (23,7%)**
Nativa	45 (76,2%)**
Protésica	14 (23,7%)**
Asociadas a dispositivo	4 (6,78%)**
Laboratorio	
Conteo leucocitos (x campo)	10090 (7480-15390)*
PCR (mg/L)	68,9 (20,6-112,3)*
Hemoglobina ingreso g/L	10,1 (8,9-11)*
Alanino aminotrasferasa (TGP) U/L	22 (15-38)*
Aspartato aminotransferasa (TGO) U/L	24 (20-41)*
Bilirrubina total mg/dL	0,57 (0,3-1,1)*
Score SOFA de ingreso	2 (1-16)*

EI: endocarditis infecciosa; PCR: proteína C reactiva; * Mediana y rango intercuartil; ** Frecuencias y porcentajes

Respecto a las comorbilidades, se observó que la mayor parte de los pacientes presentaron patología cardiaca predisponente: cardiópata congénita en un 42,3% (siendo la válvula aórtica bicúspide la más común en un 60%, seguida de la presencia de cortocircuitos en un 20%); portadores de prótesis valvulares en un 23,7% (la mayoría endocarditis protésica temprana); estenosis aortica (15,2%), portadores de marcapaso (6,7%) y cardiopatía isquémica (5,1%). Ninguno de los pacientes presentó episodios de EI previa. El 23,7% de los pacientes eran hipertensos, 11,8% tenían diagnóstico de diabetes *mellitus*,13,5% tenían diagnóstico de enfermedad renal crónica (ERC), de los cuales la mitad estaba en hemodiálisis (6,7% del total de la población).

**Figura1. Distribución:**
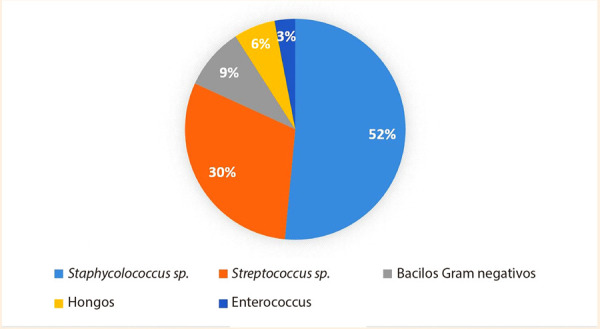
de los patógenos aislados en hemocultivos en EI complicada.

El síntoma más frecuente fue la disnea (52,5%), en segundo lugar, la astenia (38,9%) y una mínima cantidad refirió sensación de alza térmica (13,5%). Los signos encontrados durante el examen físico fueron fiebre (69,4%), presencia de un soplo cardiaco nuevo (64,4%), congestión pulmonar (15,2%) y focalización neurológica (6,7%). Dentro de los exámenes auxiliares obtenidos al ingreso se observó que el 50,8% de los pacientes presentaron leucocitosis, el 96,6% presentó valores de proteína C reactiva (PCR) elevados, con una mediana de 68,9 mg/L. A todos los pacientes se les tomaron hemocultivos a su ingreso **(**Figura 1**)**; 33 fueron positivos (55,9%), siendo el germen aislado más frecuentemente el *Staphylococcus* spp. (51,5%).

El compromiso monovalvular fue el más frecuente (76,2%), siendo la válvula aórtica la más afectada, tanto en pacientes con válvula nativa como en portadores de prótesis valvulares **(**Tabla 1**)**; también hubo afección directa de defectos congénitos en cuatro casos (6,7%) y de cables de MCP en dos pacientes (3,3%). Mediante ecocardiografía el hallazgo más común fue la presencia de vegetaciones, y la complicación más frecuente fue la perforación de velos con insuficiencia valvular **(**Tabla 2**)**; mientras que en los hallazgos intraoperatorios el más frecuente fue la vegetación (57%) y la complicación más reportada fue el absceso (28,7%). Las complicaciones intrahospitalarias se presentaron en el 72,8% **(**Tabla 3**)**.

**Tabla 2:** Hallazgos ecocardiográficos en pacientes con EI complicada

Hallazgo	n(%)
Vegetaciones	54 (91,5%)
Vegetaciones >1cm	32 (54,2%)
Absceso perivalvular	7 (11,8%)
Perforación de velos	13 (22,0%)
Ruptura de cuerdas tendineas	2 (3,3%)
Pseudoaneurismas	4(6,7%)
Fistulas	6 (10,1%)
Insuficiencia valvular	36 (61,0%)
Dehiscencia valvular	4 (6,7%)
Disfunción protésica	5 (8,4%)

**Tabla 3:** Complicaciones prequirúrgicas y posquirúrgicas de pacientes con EI complicada

Prequirúrgicas		Posquirúrgicas	
- Bloqueo AV de tercer grado - Bloqueo AV de primer grado	2 (3,3%) 15 (25,4%)	Lesión renal	13 (23,64%)
Falla cardiaca	13 (22,03%)	Choque mixto	10 (18,18%)
Embolia al sistema nervioso central	10 (16,95%)	Neumo-hemotorax	7 (12,73%)
Choque cardiogénico	9 (15,25%)	Ventilación mecánica prolongada (>7 días)	5 (9,09%)
Trombocitopenia (<150 000u/L)	7 (11,86%)	Reingreso a sala	4 (7,27%)
Daño renal	6 (10,17%)	Disfunción protésica	2 (3,64%)
Choque séptico	4 (6,78%)	Mediastinitis	2 (3,64%)

La terapia antibiótica recibida por los pacientes se inició de manera empírica hasta tener los resultados de los hemocultivos, los antibióticos más utilizados fueron los glucopéptidos (vancomicina) en un 74,6% y los aminoglucósidos en un 69,5%, así como la asociación de rifampicina en caso de endocarditis protésica temprana.

Un total de 55 pacientes (93,2%) recibieron tratamiento quirúrgico; se realizó cambio valvular en la mayoría de ellos, y en 16 (28,5%) se realizó múltiple cambio valvular. El 72,7% de pacientes tuvo complicaciones posquirúrgicas, siendo la más frecuente el daño renal (23,6%). Se obtuvo cultivo de válvula luego de la cirugía en 32 pacientes, de estos 23 fueron negativos (71,9%) y 9 fueron positivos (28,1%); en cinco casos se halló bacilos Gram negativos y en dos de ellos se halló como patógeno a *Burkholderia cepacia*, tres fueron positivos para *Staphylococcus* spp. y uno fue positivo para *Candida* sp.

La mortalidad intrahospitalaria prequirúrgica fue de 3,4% (dos casos), uno falleció por choque séptico y embolismo cerebral, a quien, por el alto riesgo quirúrgico, se desestimó la cirugía; el segundo paciente por choque séptico y hemorragia cerebral, donde igualmente se desestimó la cirugía por alto riesgo quirúrgico. La mortalidad intrahospitalaria posquirúrgica fue de 16,9% (diez casos), la mayoria también por choque séptico, lo que hace que la mortalidad global del registro sea del 20,3%.

## Discusión

En los últimos años se ha visto un gran cambio en la epidemiologia e incidencia de la EI ^2^^-^^6^. La incidencia se ha incrementado de siete casos por 100 000 personas-año en el año 2000, a 15 casos por 100 000 en el 2011 ^5^. En Latinoamérica el único país con un estudio multicéntrico sobre EI es Argentina (EIRA1,2,3) ^8^, en donde se ha visto un aumento en la incidencia de casos; en Chile se documentó en el 2008 una incidencia de 2-3 casos por 100 000 ^9^, la cual también ha ido incrementado con el transcurrir de los años ^10^. En el Perú no contamos con estudios para evaluar la incidencia de EI, sin embargo, en los últimos años se ha visto una tendencia al alza.

En el presente estudio se observó el predominio de afección del sexo masculino, resultados que concuerdan con estudios publicados ^7^^-^^11^, lo que podría explicarse por la protección que confieren los estrógenos al endotelio de las mujeres en edad reproductiva ^11^^,^^12^ Asimismo, se puede ver cómo la mediana de edad se ha incrementado en comparación a estudios previos realizados en Perú, en los que la media de edad se situaba entre 40 y 42 años ^13^^-^^15^, mientras que en nuestro estudio se encuentra alrededor de los 50 años, lo que concuerda con el aumento de media de edad que se ve en países desarrollados, en los que la media de edad ahora es de 60 años ^2^^,^^4^^,^^5^^,^^7^^,^^8^^,^^16^. En el ESC-EORP EURO-ENDO, un estudio multicéntrico que incluyó pacientes de Europa y Latinoamérica, se vio una mayor media de edad entre los países europeos en comparación con los países no europeos (60,97±17,36 vs. 52,66±19,01), resultados acordes con el presente estudio ^7^.

En cuanto a las comorbilidades, se encontró una alta incidencia de EI en pacientes con cardiopatía previa, contrario a la disminución que se ha descrito en diferentes estudios ^(7.8,10,16)^; sin embargo, esto puede relacionarse con el hecho de que nuestro centro es de referencia a nivel nacional y que la mayoría de las EI referidas eran complicadas y necesitaban tratamiento quirúrgico. Debemos resaltar que las cardiopatías congénitas y el ser portador de prótesis valvular fueron factores predisponentes frecuentes en nuestro estudio, similar a los hallazgos reportados por Cecchi E. *et al*. en el registro italiano de endocarditis infecciosa (RIEI) en el que una condición valvular predisponente fue muy común (50,37%), principalmente por la presencia de enfermedad valvular degenerativa o válvulas protésicas (26,29%) ^17^; en el EURO-ENDO se observó una baja incidencia de EI en cardiopatías congénitas (11,7%); sin embargo, se observó un aumento de la proporción de pacientes con EI portadores de válvulas protésicas (30,1%) ^7^.

Datos parecidos fueron obtenidos en Latinoamérica en los que se vio una alta proporción de pacientes con prótesis valvulares como factor predisponente ^8^^,^^11^^,^^19^; en el 2006 se encontró en el Perú que la cardiopatía de base era la causa predisponente principal en el 93,3% de los casos, encabezando la lista la enfermedad valvular reumática ^14^, la cual, en nuestra serie, no ha sido observada, situación que refleja la tendencia al descenso de esta patología como factor predisponente para EI ^7^^,^^8^^,^^11^.

El germen más frecuente fue *Staphylococcus* spp. y en segundo lugar *Streptococcus* spp, esto es similar a los hallazgos de Saito C. *et al.*^13^ en el 2014, en los que el germen mayormente aislado en los hemocultivos fue *Staphylococcus* spp. y muestra el cambio que se ha presentado en la microbiología de la EI en el Perú, ya que estudios realizados en el 2006 y 2009 se tenía como principal causante de EI al *Streptococcus* spp. en un 54,5% de los casos de hemocultivos positivos ^14^^,^^15^. El mismo cambio se ha visto internacionalmente, convirtiendo al *Staphylococcus* spp. en el germen más comúnmente aislado, especialmente *Staphylococcus aureus*, el que también es un predictor de mal pronóstico en pacientes con EI ^3^^,^^6^^,^^16^^-^^21^. En el EIRA3 también se encontró como principal agente causal a *S. aureus*, al igual que en los estudios realizados en Uruguay y Chile ^8^^,^^9^^,^^11^. El 44,1% de los cultivos fue negativo, un porcentaje muy alto en comparación a otras series internacionales, en las que se reporta un porcentaje de 8,6% (EIRA3), y 11,1% (ICE-PCS); esto se debe a que en otros países, aparte de los cultivos, se realizan pruebas moleculares para determinar el agente etiológico ^8^^,^^22^; además, como muchos de estos pacientes venían referidos de otros hospitales ya tenían tratamiento antibiótico previo, que podría haber influido en la negativización de los hemocultivos ^16^^,^^17^^,^^22^. La alta tasa de cultivos de válvula negativos podría deberse al uso de antibioticoterapia de larga data previo a la cirugía ^8^^,^^11^^,^^22^; sin embargo, lo resaltante de estos hallazgos es que los bacilos gram negativos fueron los microorganismos aislados más frecuentemente, con el 55,5% del total de los cultivos de válvula positivos.

La válvula más afectada fue la aortica, seguida de la válvula mitral, lo que muestra un cambio en comparación con los estudios previos realizados en Perú en los que la válvula más afectada era la mitral 58-62% de los casos ^13^^,^^14^. Nuestros resultados son similares a los hallados por Perez D. *et al.*^11^, en donde la principal localización fue la válvula aórtica (46,5%), seguida de la válvula mitral (23,8%); también coinciden con lo observado en el EIRA 3 (aortica 46% - mitral 33% - pulmonar 20,7%) ^8^ al igual que en el EURO-ENDO, en el que la localización más frecuente fue sobre válvula aórtica (49,5%), seguida de la válvula mitral (42%) ^7^. Esto difiere de lo hallado en el ICE-PCS, en donde predominó EI sobre válvula mitral en 41%, seguida por la infección sobre válvula aortica en 38% ^22^, al igual que en el CADRE-IE donde se observó una mayor localización de EI sobre válvula mitral (59%), seguida de válvula aortica (33,3%) ^16^. La afección multivalvular que encontramos fue relativamente mayor a otras series (EIRA3 8,4% y EURO-ENDO 18,2% de afección multivalvular) ^7^^,^^8^.

El hallazgo ecocardiográfico más reportado en este estudio fue la presencia de vegetaciones, similar a la mayoría de registros internacionales en los que las vegetaciones se reportan en más del 80% de los casos ^16^^,^^22^. Los abscesos son la complicación descrita con mayor frecuencia ^6^ y fueron las complicaciones paravalvulares más frecuentes reportadas por ICE-PCS en 15% de pacientes ^22^; en nuestro estudio se encontró que en el reporte operatorio se describen 16 casos (28,5%) de abscesos, el doble de los encontrados por ecocardiografía, lo que se asocia con la extensión y persistencia de la infección ^6^.

Una de las complicaciones más frecuentes que encontramos fue el bloqueo auriculoventricular (28,8%), el cual está asociado con la posibilidad de presentar extensión paravalvar de la infección como los abscesos ^6^; esto se vio reflejado en el hallazgo intraoperatorio de 28,5% de abscesos (10-35% en otras series) ^11^; los cuales constituyen un predictor de mortalidad para los pacientes con EI ^7^.

Hay varios factores identificados como predictores de riesgo embólico en pacientes con EI, incluso habiéndose iniciado tratamiento antibiotico: tamaño y movilidad de las vegetaciones, EI multivalvular, infección por *S. aureus* y antecedente de evento embólico ^23^; en este estudio encontramos 16,9% de casos que presentaron embolia cerebral, datos similares a los encontrados en EIRA3 en el que el 15,5% presentó eventos embólicos cerebrovasculares ^8^. Otra de las complicaciones que se presentaron previo a la cirugía fue el choque cardiogénico en 15,2% de pacientes y el choque séptico en 6,7%; Stockins B. *et al*. ^19^ reportaron que la letalidad intrahospitalaria de EI fue de 27,1%, siendo la mayoría secundarios a *shock* cardiogénico, considerándolo así un factor de mal pronóstico.

La mortalidad global en este estudio fue similar a los obtenidos en registros internacionales en los que la mortalidad varia de 15-20% ^1^^,^^3^^,^^6^^,^^7^^,^^24^; en Chile se reportó una mortalidad intrahospitalaria de 20,4% en pacientes con EI que requirieron cirugía y una sobrevida a los 10 años del 65% ^25^. En Perú se reportó una mortalidad intrahospitalaria variable del 6,1-33,3%, dependiendo de la serie revisada ^13^^-^^15^.

Nuestro estudio tiene algunas limitaciones como la escasa muestra, la falta de seguimiento a largo plazo, y el haber sido realizado en un solo centro al que solo se refieren casos complejos, por lo que no representa la verdadera epidemiología de la EI a nivel nacional.

## Conclusiones

A pesar de los avances en el diagnóstico y tratamiento, la endocarditis infecciosa continúa siendo un desafío en la práctica diaria. En este estudio encontramos que la mortalidad intrahospitalaria se mantiene alta y que la mayor dificultad se centra en un diagnóstico precoz. Nuestros resultados son comparables a los encontrados internacionalmente, los que reflejan el cambio que está sufriendo esta patología tanto en su microbiología como en su epidemiologia.
